# Greedy de novo motif discovery to construct motif repositories for bacterial proteomes

**DOI:** 10.1186/s12859-019-2686-8

**Published:** 2019-04-18

**Authors:** Hamed Khakzad, Johan Malmström, Lars Malmström

**Affiliations:** 1 Faculty of Science, Institute for Computational Science, University of Zurich, 429 Winterthurerstrasse, 190, Zurich, CH-8057 Switzerland; 2 Service and Support 430 for Science IT (S3IT), University of Zurich, Winterthurerstrasse, 190, Zurich, CH-8057 431 Switzerland; 3 Swiss Institute of Bioinformatics (SIB), Lausanne, Switzerland; 4 Division of Infection Medicine, Department of Clinical 432 Sciences, Lund University, Tornavagen, 10, Lund, SE-22184 Sweden

**Keywords:** De novo motif discovery, Infectious diseases, Group A streptococcus

## Abstract

**Background:**

Bacterial surfaces are complex systems, constructed from membranes, peptidoglycan and, importantly, proteins. The proteins play crucial roles as critical regulators of how the bacterium interacts with and survive in its environment. A full catalog of the motifs in protein families and their relative conservation grade is a prerequisite to target the protein-protein interaction that bacterial surface protein makes to host proteins.

**Results:**

In this paper, we propose a greedy approach to identify conserved motifs in large sequence families iteratively. Each iteration discovers a motif de novo and masks all occurrences of that motif. Remaining unmasked sequences are subjected to the next round of motif detection until no more significant motifs can be found. We demonstrate the utility of the method through the construction of a proteome-wide motif repository for Group A Streptococcus (GAS), a significant human pathogen. GAS produce numerous surface proteins that interact with over 100 human plasma proteins, helping the bacteria to evade the host immune response. We used the repository to find that proteins part of the bacterial surface has motif architectures that differ from intracellular proteins.

**Conclusions:**

We elucidate that the M protein, a coiled-coil homodimer that extends over 500 A from the cell wall, has a motif architecture that differs between various GAS strains. As the M protein is known to bind a variety of different plasma proteins, the results indicate that the different motif architectures are responsible for the quantitative differences of plasma proteins that various strains bind. The speed and applicability of the method enable its application to all major human pathogens.

**Electronic supplementary material:**

The online version of this article (10.1186/s12859-019-2686-8) contains supplementary material, which is available to authorized users.

## Background

The rise of antibiotics resistant bacteria poses a major global health issue predicted to cause 10 million deaths per year in 2050, more than heart disease and cancer combined [1]. The increasing resistance to antibiotics necessitates the development of alternative treatment strategies. One promising alternative treatment strategy includes the disruption of protein binding interfaces between bacteria and human proteins to disarm bacterial defense systems [2]. Such strategies require high-confident identification of sequence motifs that correspond to a structural unit that are necessary for protein folding or binding of ligands and other proteins.

Motifs are short segments of a protein sequence which shows a level of conservation throughout a protein family and beyond. Conserved motifs can be extracted from multiple sequence alignment of proteins with similar functions in different species. While finding such motifs can provide insights for prediction of functional residues, identifying and understanding them is fundamental to discovering binding interfaces in protein complexes [3]. It is generally believed that the binding interfaces forming interactions to help bacteria evade the immune system or to obtain nutrients are comparatively more conserved compared to interactions that are benefiting the host, such as surface exposed epitope. Over time, this results in segments of exposed proteins that are significantly more conserved for functional reasons.

Disrupting the protein-protein interactions by targeting the conserved segments would potentially facilitate the host immune response [4–6]. However, the high variability of bacterial surface proteins makes it challenging to study them with traditional sequence analysis methods. InterPro for example [7] contains motifs for the anchor and the signal peptide whereas the rest of the protein sequence remains largely unannotated. Multiple-sequence alignment algorithms typically run into problems with the variable number of repeats and tends to produce highly gapped alignments. The rapid growth of known bacterial protein sequences presents an opportunity to identify protein-family specific motifs (in contrast to Interpro that attempts to find motifs common to multiple families).

Group A streptococcus (GAS) is one of the most important bacterial pathogens causing over 700 million mild infections such as tonsillitis, impetigo and erysipelas and, occasionally, severe invasive infections including sepsis, meningitis or necrotizing fasciitis with mortality rates up to 25% [8]. Surface proteins play important roles in the interaction with host proteins [9]. Several bacterial surface proteins interact with numerous of host proteins, forming complex protein-protein interaction networks.

One of the key surface proteins of S. pyogenes is the M protein, a coiled-coil homodimer that extends over 500 Å from the cell wall. The M protein is capable of binding several plasma proteins such as fibrinogen [6] and albumin [10, 11]. A crystal structure of M and fibrinogen was published in 2011 demonstrates that the M and fibrinogen form a cross-like complex structure. Further, the M protein is composed of several repeats that are present a variable number of times; some of these repeats overlap with protein-protein interactions binding interfaces [12–15]. Accordingly, a comprehensive repository of the motifs in coiled-coil proteins and their relative conservation grade is a prerequisite to target the protein-protein interaction that bacterial surface protein makes to host proteins [16].

Here, we present a strategy to iteratively identify protein-family specific motifs from large genome resources, then mask all occurrences of these motifs until no more significant motifs can be found. We applied this strategy to a GAS strain as a model system. We constructed a compendium of almost 60000 motifs for GAS. Further, we demonstrate the power of the approach using the M protein and describe the motif resource in general terms.

## Methods

### Outline of the algorithm

**Fig. 1:**
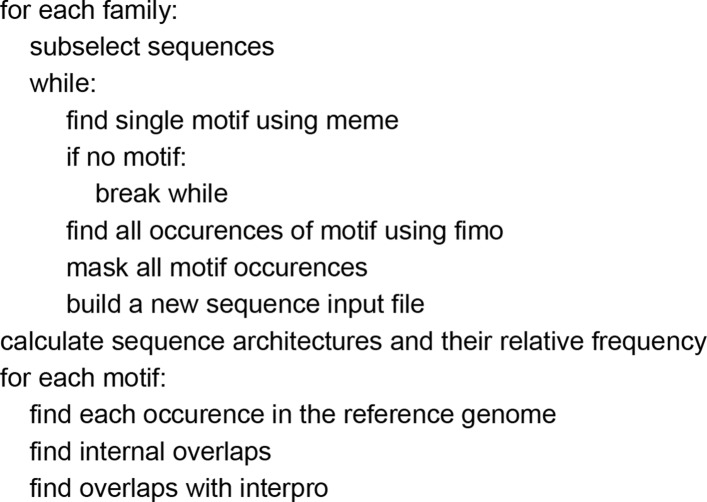
The pseudo-algorithm of de novo motif discovery approach

The algorithm starts with a database of protein sequence families and sub-selects a user-defined number (here 100) of sequences for each family containing more than 100 sequences as shown in the pseudocode in Fig. 1. The main part of the algorithm finds the first motif in a sequence and mask all identified occurrences, then remove them from the sequence and produce one new sequence for each occurrence in the second iteration. After finishing this loop, all identified motifs are stored in the main repository and followed by architectural analysis by considering the occurrences of each motif in the entire genome and by computing the internal overlaps of that motif inside the family as well. In the last step, the results will be compared with InterPro to find common overlapping motifs to report and the new ones for further analysis. All the results as well as the main repository of all discovered motifs are stored and available in a SQLite table. SQLite is an embedded SQL database engine that implements a transactional SQL database engine. The code for SQLite is in the public domain and free for use.

### Construction of protein families

**Fig. 2:**
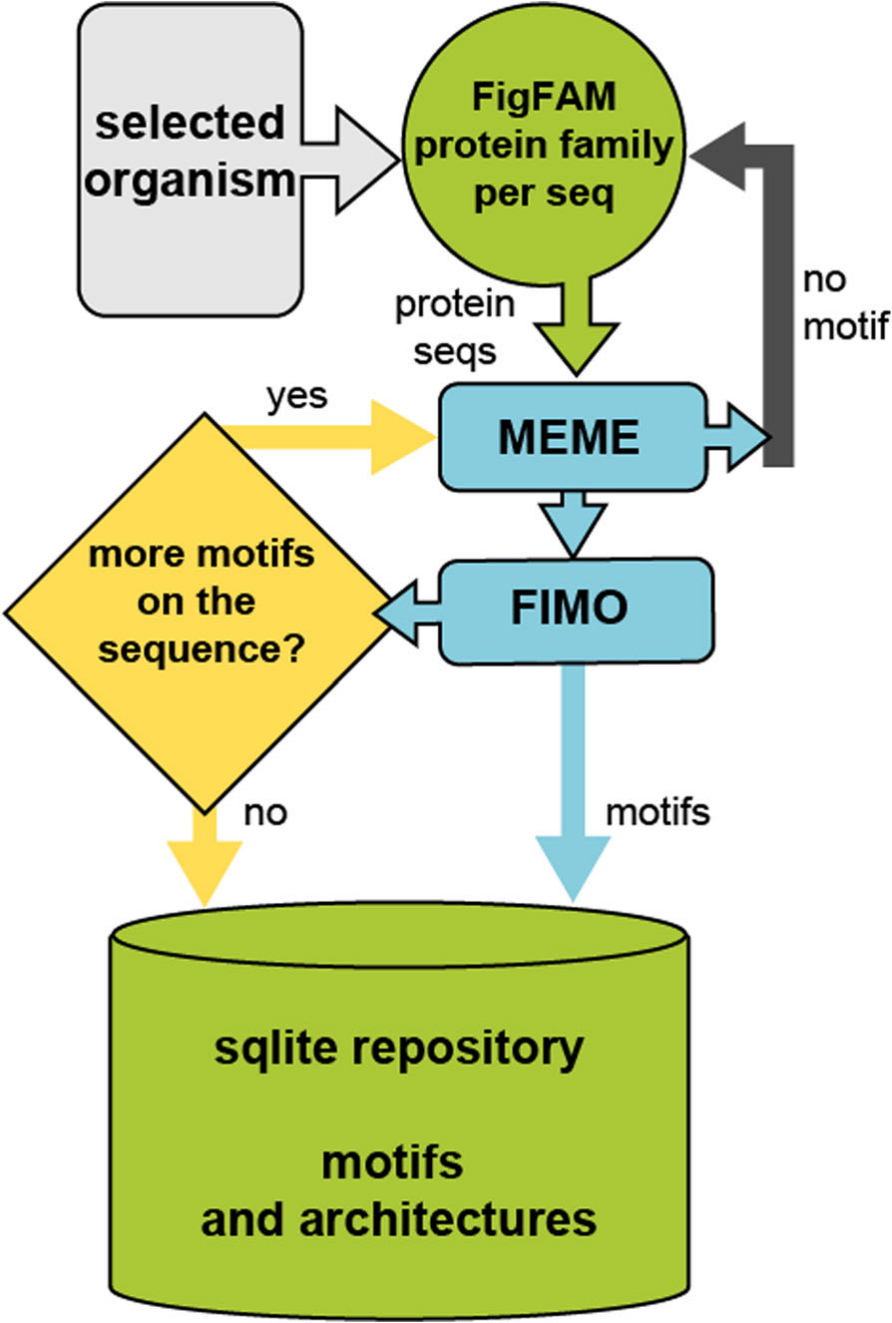
The workflow of de novo motif discovery approach. All FIGfams families were downloaded for a user-specified organism and get through the core of the processing -MEME and FIMO- where all the motifs are discovered and masked in an iterative process. A sqlite repository stores all the motifs and motif architectures with required information like organism name, FIGfams family name, the start and stop points on the sequence and etc

We selected a representative genome from an invasive M1 S. pyogenes isolated in Ontario, Canada. This sample is available with id 293653.4 from PatricBRC, the bacterial bioinformatics resource database [17]. This genome has 1931 coding sequences (CDS). We downloaded additional 70459 genomes from PatricBRC. This number here refers to the number of genomes available at PatricBRC that had both an.ffa protein fasta file and.cds files that contains a table which links the PatricBRC sequence accession number to the FigFam ID [18]. We used this resource to build one protein fasta file per FigFam ID filtering out duplicate entries. We constructed 1564 FIGfams families containing a total of 9,041,083 protein sequences of which 3,817,065 were unique at the amino acid level. This sequence resource was used as input to the workflow outlined in Fig. 2.

### MEME and FIMO

Figure 2 shows the general workflow of our approach, where we make use of MEME [19, 20] and FIMO [21] in the core part of the system to handle motif discovery and masking the multiple occurrences of each motif on the sequence. MEME is an open-source application which has been widely used for sequence motif discovery and analysis in both DNA and proteins. It is based on GLAM2 algorithm [22] and enables covering of motifs containing gaps. While MEME finds a single occurrence of a motif in the sequence, FIMO is able to consider the MEME’s output and define multiple occurrences for any individual gapped or un-gapped motifs. FIMO assign different scores for each matched sequence according to a dynamic programming approach [23] and then motif-specific q-values are computed based on a bootstrap procedure [24]. FIMO’s outputs are considered according to their p-values, and q-values make it possible to set a user-defined thresholds to cover only specific motif occurrences.

### InterPro

InterProScan [7] is a reference resource that provides a functional analysis of protein sequences by classifying them into families and predicting the presence of domains and important sites. In order to achieve a general view of the coverage of our approach, we compared the generated de novo based motif repository of GAS with all GAS-related motifs in InterProScan.

### Assigning proteins to cellular compartments

All proteins were assigned to one of seven compartments by using information from mass spectrometry experiments, annotations from several databases followed by manual curation. In short, we identified exposed, cell wall associated and secreted protein using data from Karlsson et al. [9]. Transmembrane proteins were identified using TMHMM [25]. DNA associated protein and transcription factors were identified using InterPro [7] and RegPrecise [26]. All other proteins were assigned to the intracellular compartment.

### Software availability

MEME and FIMO 4.11.1 was used through out the project. The workflow is implemented in GC3pie [27] which makes it possible to parallelize over all available computational cores. All parts of the workflow are written in Python 2.7 and is wrapped by applicake, an open-source and free framework useful when designing workflows. The workflow is available through a singularity container [28] and the container together with the data and an ipython notebook contains instructions and examples to parse the data are provided online with this DOI: 10.5281/zenodo.1403142.

## Results

### Sub-selection

**Fig. 3:**
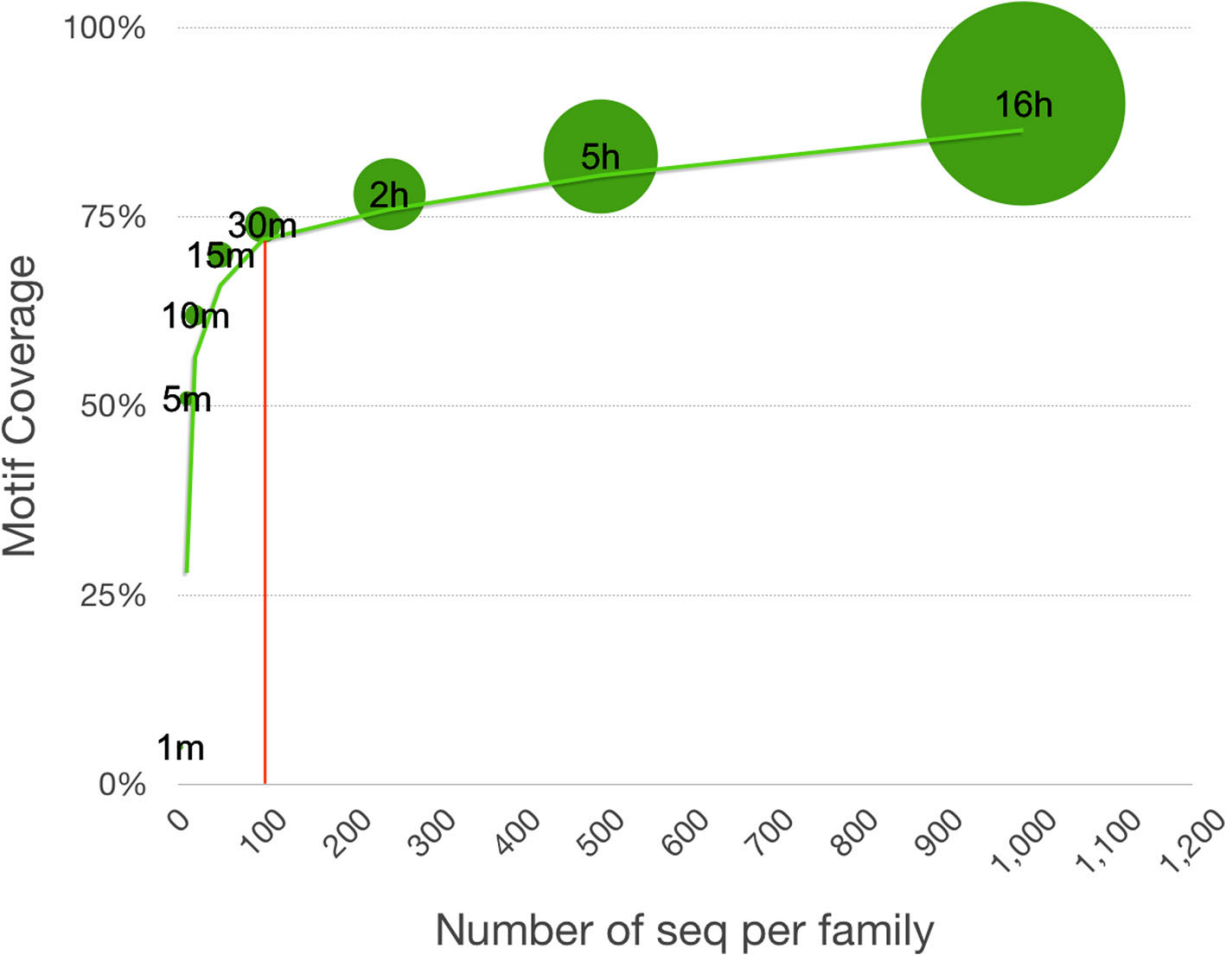
Sub-selection test on two sample families. Two sample families are selected and analyzed by sub-selection test. The bubble graph indicates that selection of 100 sequences has a good coverage while saving computational resources more than 20-fold

We analyzed a large sequence database of all GAS proteins containing 1564 FIGfams sequence families as outlined in the Methods section. The FIGfams contain a different number of sequences. This begs the question whether a subset of them would be sufficient to cover most of the motifs. We designed a general sub-selection test to reduce the number of sequences due to computational resource reasons. The sub-selection considers two different families and select a set of 2, 10, 20, 50, 100, 250, 500, and 1000 sequences randomly and repeat the whole analysis for 10 times. In each sub-selection test, we ran the workflow to find all the motifs, and we made an average of motif-coverage between all 10 repeats. Figure 3 demonstrates that sub-selection of 100 sequences is sufficient to cover the majority number of all motifs while reducing time and computational resources more than 20-fold (see Table 1).

**Table 1:** Comparison of sub-selection test between different families with different number of selected sequences

Num of sequences	Coverage	Computational time
2	5%	1m
10	51%	5m
20	62%	10m
50	70%	15m
100	74%	30m
250	78%	2h
500	83%	5h
1000	90%	16h
> 2000	∼ 100%	> 2d

The number of sequences, the motif coverage in percentage (which is the average of 10 repeated test) and the computational time on 1 CPU are shown

### MEME/FIMO

**Fig. 4:**
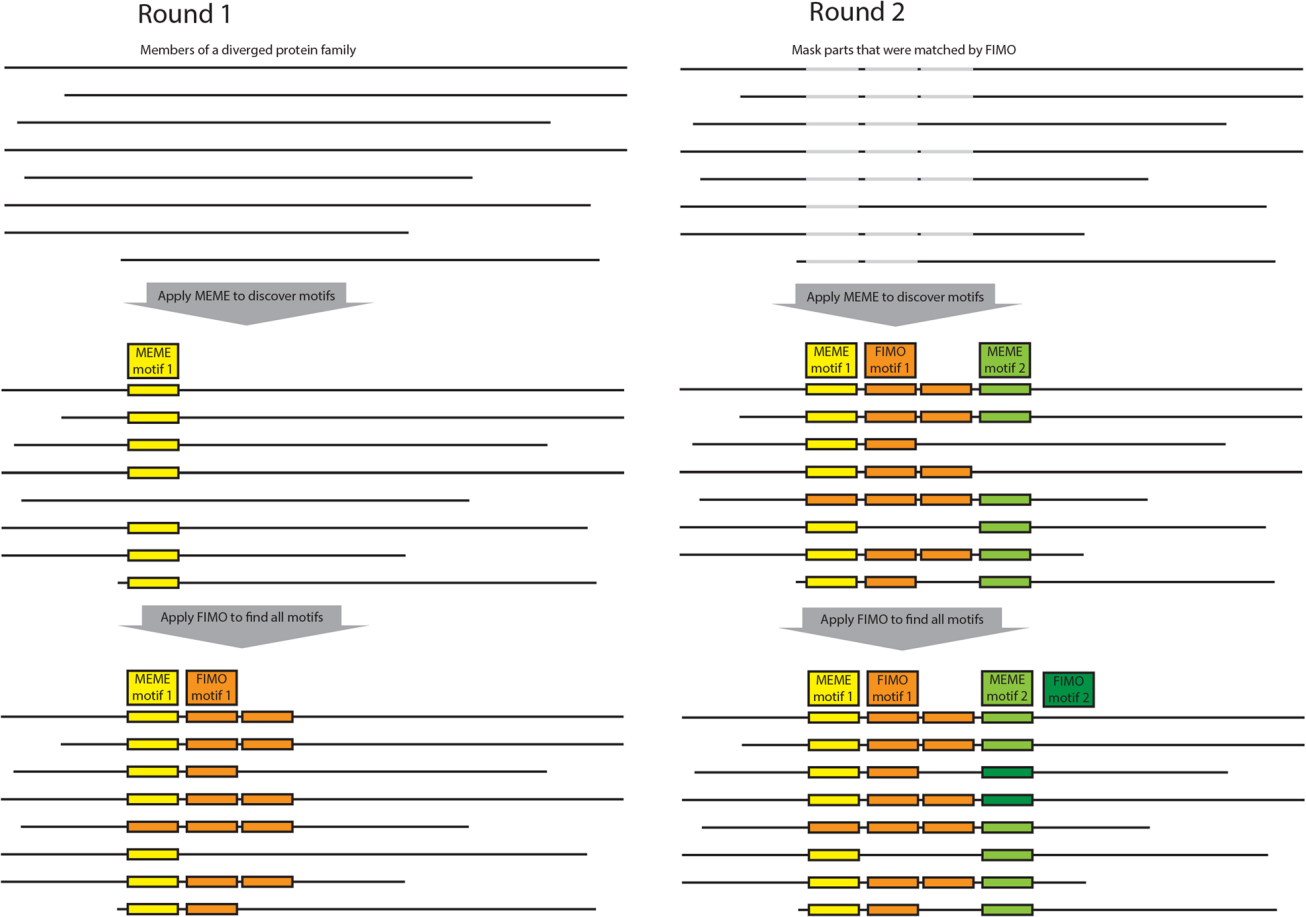
Two rounds (first to the left, second to the right) of the algorithm is displayed. It starts with a collection of sequences and then discovers motifs in this collection using MEME. It uses FIMO to find additional occurrences of the motif within the sequence collection. In the second round, the motifs are masked (gray bars) before MEME is applied once more. The algorithm iterates through round 3 to N until no more motifs are found, or the sequence collection is fully annotated

The workflow starts by entering the name of the desired organism and the q-value cut-off (optional) which are the only required inputs (Fig. 2). In the second phase, all FIGfams protein families related to the input organism are downloaded and stored in a database. Then, by considering the accessibility of computational resources, de novo motif discovery on protein families starts. Figure 4 shows two sample runs of the algorithm where MEME is applied to the sequence collection, restricting the number of identified motifs to one. Motif occurrences were discovered in the sequence collection using FIMO, and only occurrences with e-values of 1e^-6^ or lower were considered. The proteins were split using the number of occurrences and remaining parts longer than ten amino acids are carried forward to create a new merged sequence collection, mixed with full-length and partial proteins.

The new sequence collection is used as the input for each iterative round of MEME, FIMO, split until no more significant motifs could be discovered, or all remaining sub-sequence were below ten amino acids. All the motif occurrences with corresponding features are stored in an SQLite database. To give further information to the user, known motifs are also integrated from [10] and the InterPro database and visualized using pViz.js [29].

### Protein M1 Motif discovery

As an example of application on specific protein family, we collected a large sequence collection of M proteins from four sources: PatricBRC, genomes we have previously sequenced and assembled [30], the M database from CDC (Centers for Disease Control and prevention) and the UniProtKB/TrEMBL database. Any M protein sequence without motifs representing an anchor or a signal peptide was discarded, and the remaining sequences were reduced to a 98% sequence identity using CD-HIT [31]. In total, the algorithm ended after 18 rounds, resulting in 20 motifs from the M protein sequence collection. The SF370 M1 protein reference [32] contained motifs m01-m03, m05-m08, m11-m14, m16-m17 but not m04, m09-10, m15, and m18-20. Additional file 1 contains the logo of all discovered motifs.

**Fig. 5:**
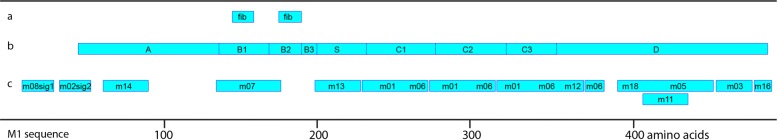
The auto-generated result of our approach on M1 protein. a: Binding interfaces of fibrinogen according to the reference crystal structure (PDB id 2XNX). [6]. b: M1 domains proposed in [10]. c: The output results of our approach

Figure 5 shows the general motif architecture as the output of the algorithm. Note that an architecture (motif pattern) shows the distribution of motifs over the entire protein family. By considering such representation, it is possible to show the general motif pattern that most of the proteins in the family follow. So, the architectural motif view helps to find potential protein-protein interaction binding sites as the majority member of the family desire to follow such pattern. Accordingly, we found a total of 123 motif architectures, and of these, 85% (104) are associated with a single serotype.

Here in M protein, architecture [m01:3, m02:1, m03:1, m05:1, m06:4, m07:3, m08:1, m11:1, m12:1, m14:1, m16:1, m17:1] is the architecture that exists in several serotypes (emm52, emm23, emm16, emm83, emm10). For the M1 proteins, we identified seven motif architectures (Table 2). For emm1 in Fig. 5, we see that m08 and m02 are the first and second part of the YSIRK signal peptide. m03 largely overlap with the anchor region. m01 and m06 correspond to the C repeats, m07 overlaps with the B repeats although we fail to identify the second and third B-domain. m13 finds the S region and m14 overlap partly with the A domain. The D domain is largely split into several motifs - m12, m18 followed by m05.

**Table 2:** Architectures identified for M1 protein

Architectures					Support
1	m07:3	m01:1	m06:2	m05:1	1
2	m07:3	m01:2	m06:4	m05:1	1
3	m07:3	m01:3	m06:4	m05:0	1
4	m07:3	m01:4	m06:5	m05:1	1
5	m07:3	m01:2	m06:3	m05:1	2
6	m07:2	m01:3	m06:4	m05:1	2
7	m07:3	m01:3	m06:4	m05:1	10

Seven different motif architectures are identified with different level of supports. The last one (most important one) supports by 10 proteins in the family. The table also shows that motifs m07, m01, m06, and m05 are the most prevalent motifs in M1 and also generally in M protein family

### Analysis of conserved motif in GAS genome

We evaluated identified motifs separately based on protein families in different cellular compartments (Table 3). The main idea is to provide a general comparison between protein families in different cellular compartments in terms of motif-based conservation grade which helps to discover the general evolutionary pressure on cellular compartments and further distinguishing potential drug targets inside and outside the cell. To do that, one should consider the fact that the number of motifs in each compartment is a function of sequence length and the average is dependent on sequence variability. Such dependency affects the comparison between different protein families led to results that are biased against sequence length. To address these issues, we represent motif architecture per sequence and most importantly per family. Accordingly, each protein or its related family can have one or several architectures based on motif variability on that family. Consequently, protein families with few architectures indicates higher sequence conservation inside the family and generally shows that the family has more conserved motifs to do special cellular functions. In this way, by comparing the number of architecture in two different protein families, it is possible to state which family is more conserved.

**Table 3:** Protein characterization in different cellular compartments

	Compartments	Number of sequences
0	DNA	104
1	Exposed	40
2	Intracellular	1172
3	Secreted	52
4	Transcription factor	71
5	Transmembrane	217
6	Cell wall	130

**Fig. 6:**
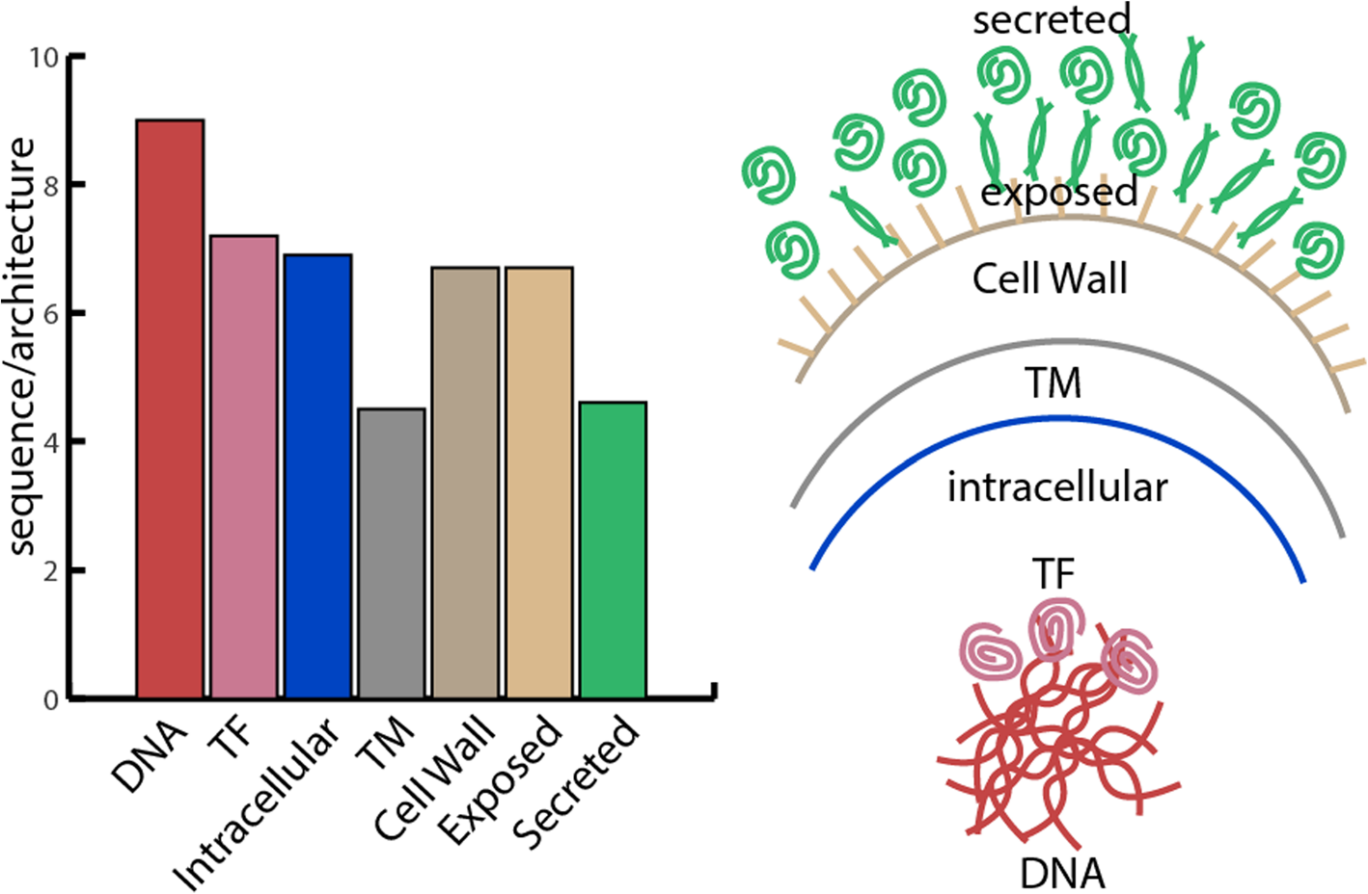
Motif-based architectural comparison between different cellular compartments. The bar-plot to the left shows the general comparison while different cellular compartments are separated in a schematic cell view to the right. Transmembrane proteins (TM) and secreted proteins have the least number of architecture per family. It shows that motif diversity in these compartments are high and changing by the time. In contrary DNA-related proteins and Transcription Factors (TF) show more conserve motifs on their sequence with having the highest number of architecture per family

As shown in Table 4, most variable proteins in GAS are transmembrane and secreted proteins which are less conserved and have a more diversified interaction with host proteins. Most conserved proteins are DNA-related and transcription factors together with intracellular proteins that have special machinery roles inside the cell. Transmembrane proteins which play crucial role as the transportation system on the bacterial surface are also more evolved according to the evolutionary pressure. In general as Fig. 6 indicates, we can conclude that the evolutionary pressure is lower on intracellular proteins compared to the surface and secreted proteins.

**Table 4:** Comprehensive comparison based on number of motifs per sequence and architecture in different cellular compartments

Compartments	cds	Unique sequences	Motifs	Motifs/sequences	Sequences/architectures
DNA	96	22	86	3.91	9.04
Exposed	97	17	92	5.41	6.70
Intracellular	2116	304	1273	4.19	6.94
Secreted	204	23	193	8.40	4.65
Transcription factor (TF)	208	35	127	3.63	7.15
Transmembrane (TM)	714	75	601	8.01	4.55
Cell wall	407	60	284	4.73	6.69

### Comparison with InterPro

To compare our results to InterPro, we analyzed and filtered motifs based on their signature from InterProScan which revealed that 11996 distinct motifs related to GAS are not recognized/discovered by InterProScan (71.15% of all discovered motifs) while there are many important motifs also in common (28.85%). Table 5 contains the list of most commonly overlapping motifs with special InterPro description which discovered by our approach.

**Table 5:** The InterProScan results that are most commonly overlapping with a motif

Num	interpro_ac	Count	Distinct motif	Interpro description
0	None	58604	11996	None
1	IPR003439	9652	402	ABC transporter-like
2	IPR027417	6384	1187	P-loop containing nucleoside triphosphate hydr...
3	IPR003593	5060	446	AAA+ ATPase domain
4	IPR017871	1683	49	ABC transporter, conserved site
5	IPR000515	1131	178	ABC transporter type 1, transmembrane domain M...
6	IPR035906	835	204	MetI-like superfamily
7	IPR001789	750	62	Signal transduction response regulator, receiv...
8	IPR030679	498	155	ABC-type amino acid transport system, ATPase c...
9	IPR036188	392	153	FAD/NAD(P)-binding domain superfamily
10	IPR005670	356	98	Phosphate transport system permease protein 1
11	IPR036388	323	191	Winged helix-like DNA-binding domain superfamily
12	IPR036890	318	110	Histidine kinase/HSP90-like ATPase superfamily
13	IPR013785	316	274	Aldolase-type TIM barrel
14	IPR000524	307	17	Transcription regulator HTH, GntR
15	IPR003594	305	69	Histidine kinase/HSP90-like ATPase
16	IPR000843	299	22	LacI-type HTH domain
17	IPR002347	288	42	Short-chain dehydrogenase/reductase SDR
18	IPR036291	276	177	NAD(P)-binding domain superfamily
19	IPR000795	269	45	Transcription factor, GTP-binding domain
20	IPR017853	263	169	Glycoside hydrolase superfamily
21	IPR001650	237	68	Helicase, C-terminal
22	IPR014001	230	95	Helicase superfamily 1/2, ATP-binding domain
23	IPR029063	230	205	S-adenosyl-L-methionine-dependent methyltransf...
24	IPR001360	227	47	Glycoside hydrolase family 1
25	IPR036390	214	113	Winged helix DNA-binding domain superfamily
26	IPR006047	212	68	Glycosyl hydrolase, family 13, catalytic domain
27	IPR011006	209	77	CheY-like superfamily
28	IPR020846	206	92	Major facilitator superfamily domain
29	IPR001638	202	48	Solute-binding protein family 3/N-terminal do...

## Discussion

Conserved protein sequence domains, also referred to as motifs, play an important role in protein function, protein structure and protein-protein interactions. Motifs are the results of several evolutionary processes where, for example, a part of a protein is evolving at a different rate compared to other parts of the same protein. Identifying motifs are fundamental to understanding protein function and to discover putative binding interfaces.

Motifs can both be used to shed light on the evolutionary process underpinning the development of a protein family with respect to the protein’s function over time; it can also be used to produce a simplified view on the protein as a series of conserved motifs that together specify a proteins motif architecture. Although several approaches have been developed to address motif discovery on protein sequences, most are either focused on a given motif or finding motifs, such as signal peptides, that can be found in a general population of protein sequences.

Here, we developed a de novo motif discovery approach and applied to protein families that share a common ancestral protein; this resulted in a repository of motifs over an entire organism. This approach is focused on understanding the evolutionary processes that have acted on that protein family in a comparatively short evolutionary time. We developed and designed this approach as a software package which is written in Python and distributed via singularity containers [28] making it easy to install and use. We demonstrated the approach on GAS, an important human pathogen with a mortality rate of 25% at invasive infections. We also characterized the proteome-wide motif repository by comparing it to InterPro; furthermore, we analyzed the motif architecture for these proteins and discovered that the number of sequence per architecture is different for different cellular compartments.

Given the speed and flexibility of our approach, we believe it will be useful in breaking analyzing surface protein of pathogens as these proteins are under high selective pressure and therefore cannot be analyzed using more traditional approaches such as multiple-sequence alignments (MSAs). Our attempts to use various MSA algorithms failed due to high sequence variability in regions between motifs and the varying number of motifs. Also, motif searching approaches failed and only identified a small subset of the motifs that our approach discovered.

## Conclusion

In this paper, we demonstrate a proof-of-principle approach to parsing large sequence families into motifs using a denovo-based greedy approach. This simple approach can easily handle situations where parts of proteins are repeated or re-arranged, and this can be time-consuming using other approaches. While this general approach can be applied to any bacteria, we used GAS as a model system to make a comprehensive motif repository of its proteins. We further analyzed M1 protein, one of the most important virulence factor of S. pyogenes to show the motif-based architectural analysis. We observe that we over-parse some domains, but also observe that many of these large domains are only partly conserved over the sequence collection. The result indicates that many of the newly discovered motifs are not always present together with adjacent motifs, indicating that they might have different and independent functions. Interestingly, many of our newly discovered motifs are not found in any of the emm1 strains, and some of these might be responsible for binding other ligands.

## Additional file

Additional file 1 **Supplementary Table 1**. The logo of all 20 motifs obtained from our approach for M protein family is listed in a multi-page table in the supplementary material. (PDF 1075 kb)

## Abbreviations

CDC: Centers for disease control and prevention
CDS: Coding sequences
GAS: Group A Streptococcus
MSA: Multiple-sequence alignments
TM: Transmembrane
TF: Transcription factor

## References

[1] O’Neill J. Antimicrobial resistance: tackling a crisis for the health and wealth of nations. Rev Antimicrob Resist. 2014.

[2] Forthal DN. Functions of antibodies. Microbiol Spectr. 2014; 2(4). PMC4159104 25215264

[3] Bork Peer, Koonin Eugene V (1996). Protein sequence motifs. Current Opinion in Structural Biology.

[4] Ghosh Partho (2018). Variation, Indispensability, and Masking in the M protein. Trends in Microbiology.

[5] Sandin Charlotta, Carlsson Fredric, Lindahl Gunnar (2006). Binding of human plasma proteins to Streptococcus pyogenes M protein determines the location of opsonic and non-opsonic epitopes. Molecular Microbiology.

[6] Macheboeuf P, Buffalo C, Fu C-y, Zinkernagel AS, Cole JN, Johnson JE, Nizet V, Ghosh P (2011). Streptococcal m1 protein constructs a pathological host fibrinogen network. Nature.

[7] Jones P., Binns D., Chang H.-Y., Fraser M., Li W., McAnulla C., McWilliam H., Maslen J., Mitchell A., Nuka G., Pesseat S., Quinn A. F., Sangrador-Vegas A., Scheremetjew M., Yong S.-Y., Lopez R., Hunter S. (2014). InterProScan 5: genome-scale protein function classification. Bioinformatics.

[8] Mitchell TJ (2003). The pathogenesis of streptococcal infections: from tooth decay to meningitis. Nat Rev Microbiol.

[9] Karlsson C, Malmström L, Aebersold R, Malmström J (2012). Proteome-wide selected reaction monitoring assays for the human pathogen streptococcus pyogenes. Nat Commun.

[10] Akesson P, Schmidt KH, Cooney J, Björck Larsrck L (1994). M1 protein and protein h: Iggfc- and albumin-binding streptococcal surface proteins encoded by adjacent genes. Biochem J.

[11] Hauri S, Khakzad H, Happonen L, Teleman J, Malmström J, Malmström L. Rapid determination of quaternary protein structures in complex biological samples. Nat Commun. 2019; 10(1):192. 10.1038/s41467-018-07986-1. PMC6331586 30643114

[12] McMillan D.J., Drèze P. -A., Vu T., Bessen D.E., Guglielmini J., Steer A.C., Carapetis J.R., Van Melderen L., Sriprakash K.S., Smeesters P.R. (2013). Updated model of group A Streptococcus M proteins based on a comprehensive worldwide study. Clinical Microbiology and Infection.

[13] Buffalo CZ, Bahn-Suh AJ, Hirakis SP, Biswas T, Amaro RE, Nizet V, Ghosh P. Conserved patterns hidden within group a streptococcus m protein hypervariability are responsible for recognition of human c4b-binding protein. Nat Microbiol. 2016; 1:16155. 10.1038/nmicrobiol.2016.155. PMC5014329 27595425

[14] Dale James B., Smeesters Pierre R., Courtney Harry S., Penfound Thomas A., Hohn Claudia M., Smith Jeremy C., Baudry Jerome Y. (2017). Structure-based design of broadly protective group a streptococcal M protein-based vaccines. Vaccine.

[15] Sanderson-Smith Martina, De Oliveira David M. P., Guglielmini Julien, McMillan David J., Vu Therese, Holien Jessica K., Henningham Anna, Steer Andrew C., Bessen Debra E., Dale James B., Curtis Nigel, Beall Bernard W., Walker Mark J., Parker Michael W., Carapetis Jonathan R., Van Melderen Laurence, Sriprakash Kadaba S., Smeesters Pierre R. (2014). A Systematic and Functional Classification of Streptococcus pyogenes That Serves as a New Tool for Molecular Typing and Vaccine Development. The Journal of Infectious Diseases.

[16] Sjöholm K, Kilsgård O, Teleman J, Happonen L, Malmström L, Malmström J (2017). Targeted proteomics and absolute protein quantification for the construction of a stoichiometric host-pathogen surface density model. Mol Cell Proteome.

[17] Wattam AR, Davis JJ, Assaf R, Boisvert S, Brettin T, Bun C, Conrad N, Dietrich EM, Disz T, Gabbard JL, Gerdes S, Henry CS, Kenyon RW, Machi D, Mao C, Nordberg EK, Olsen GJ, Murphy-Olson DE, Olson R, Overbeek R, Parrello B, Pusch GD, Shukla M, Vonstein V, Warren A, Xia F, Yoo H, Stevens RL (2017). Improvements to patric, the all-bacterial bioinformatics database and analysis resource center. Nucleic Acids Res.

[18] Meyer Folker, Overbeek Ross, Rodriguez Alex (2009). FIGfams: yet another set of protein families. Nucleic Acids Research.

[19] Bailey TL, Boden M, Buske FA, Frith M, Grant CE, Clementi L, Ren J, Li WW, Noble WS (2009). Meme suite: tools for motif discovery and searching. Nucleic Acids Res.

[20] Bailey TL, Elkan C (1994). Fitting a Mixture Model by Expectation Maximization to Discover Motifs in Biopolymers, vol. 2.

[21] Grant CE, Bailey TL, Noble WS (2011). Fimo: scanning for occurrences of a given motif. Bioinformatics.

[22] Frith Martin C., Saunders Neil F. W., Kobe Bostjan, Bailey Timothy L. (2008). Discovering Sequence Motifs with Arbitrary Insertions and Deletions. PLoS Computational Biology.

[23] Staden Rodger (1994). Staden: Searching for Motifs in Nucleic Acid Sequences. Computer Analysis of Sequence Data.

[24] Storey John D. (2002). A direct approach to false discovery rates. Journal of the Royal Statistical Society: Series B (Statistical Methodology).

[25] Krogh Anders, Larsson Björn, von Heijne Gunnar, Sonnhammer Erik L.L (2001). Predicting transmembrane protein topology with a hidden markov model: application to complete genomes11Edited by F. Cohen. Journal of Molecular Biology.

[26] Novichkov PS, Laikova ON, Novichkova ES, Gelfand MS, Arkin AP, Dubchak I, Rodionov DA (2010). Regprecise: a database of curated genomic inferences of transcriptional regulatory interactions in prokaryotes. Nucleic Acids Res.

[27] Maffioletti S, Murri R. Gc3pie: A python framework for high-throughput computing. Proc EGI Community Forum 2012/EMI Second Tech Conf (EGICF12-EMITC2). 2012:143.

[28] Kurtzer Gregory M., Sochat Vanessa, Bauer Michael W. (2017). Singularity: Scientific containers for mobility of compute. PLOS ONE.

[29] Mukhyala K, Masselot A (2014). Visualization of protein sequence features using javascript and svg with pviz.js. Bioinformatics.

[30] Malmström Lars, Bakochi Anahita, Svensson Gabriel, Kilsgård Ola, Lantz Henrik, Petersson Ann Cathrine, Hauri Simon, Karlsson Christofer, Malmström Johan (2015). Quantitative proteogenomics of human pathogens using DIA-MS. Journal of Proteomics.

[31] Fu L, Niu B, Zhu Z, Wu S, Li W (2012). Cd-hit: accelerated for clustering the next-generation sequencing data. Bioinformatics.

[32] Ferretti J. J., McShan W. M., Ajdic D., Savic D. J., Savic G., Lyon K., Primeaux C., Sezate S., Suvorov A. N., Kenton S., Lai H. S., Lin S. P., Qian Y., Jia H. G., Najar F. Z., Ren Q., Zhu H., Song L., White J., Yuan X., Clifton S. W., Roe B. A., McLaughlin R. (2001). Complete genome sequence of an M1 strain of Streptococcus pyogenes. Proceedings of the National Academy of Sciences.

