# Supplementary material for: Greedy de novo motif discovery to construct motif repositories for bacterial proteomes
Source: BMC Bioinformatics. 2019 Apr 18;20(Suppl 4):141. doi: 10.1186/s12859-019-2686-8 (PMC6471678; doi:10.1186/s12859-019-2686-8)
Supplement: Supplementary file 1 — Supplementary Table 1. The logo of all 20 motifs obtained from our approach for M protein family is listed in a multi-page table in the supplementary material. (PDF 1075 kb) [file 12859_2019_2686_MOESM1_ESM.pdf]

Motif	Logo
m01	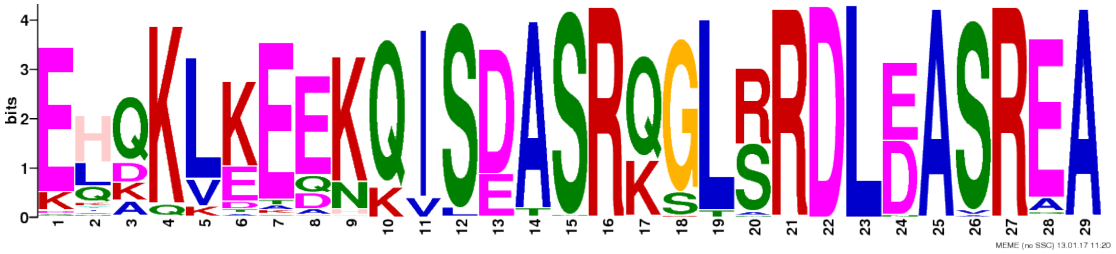
m02	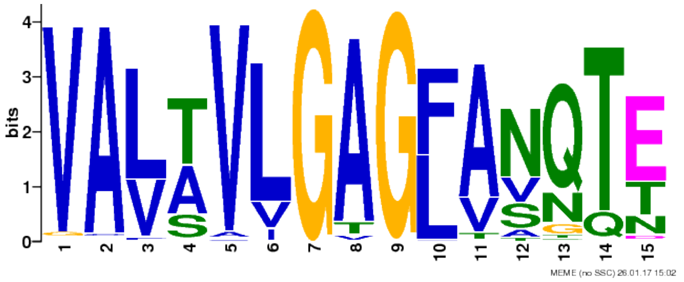
m03	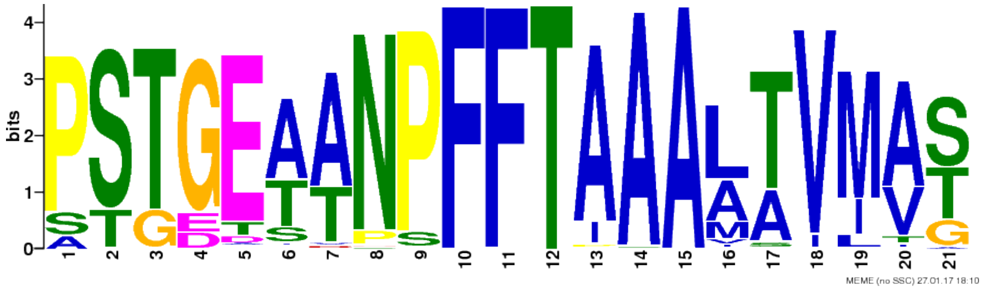
m04	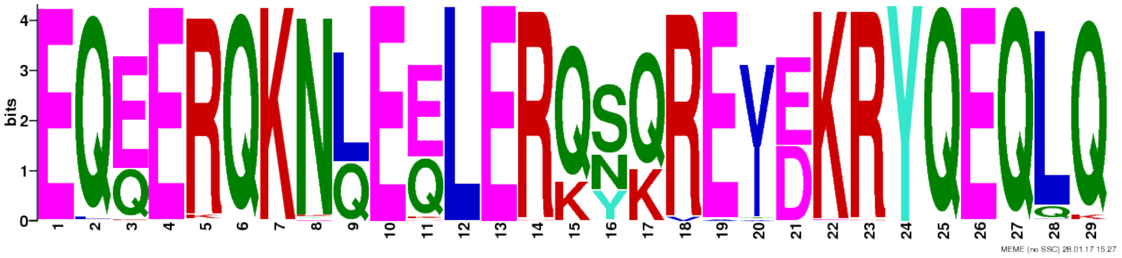
m05	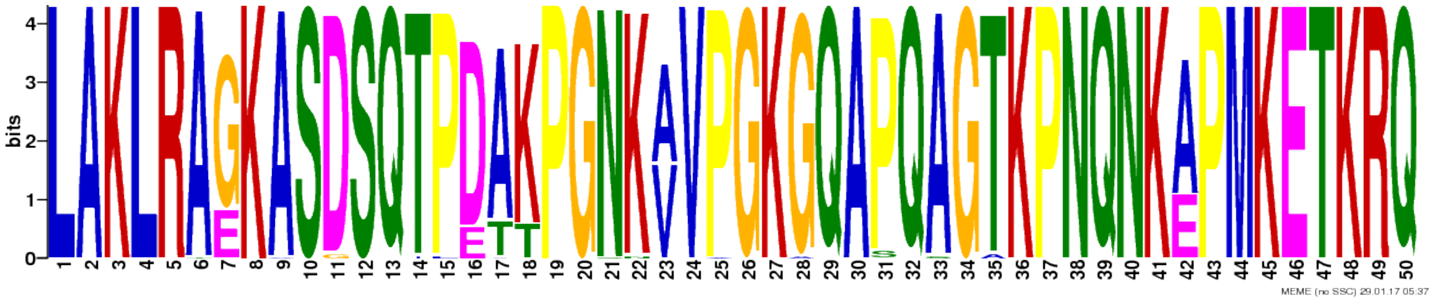
m06	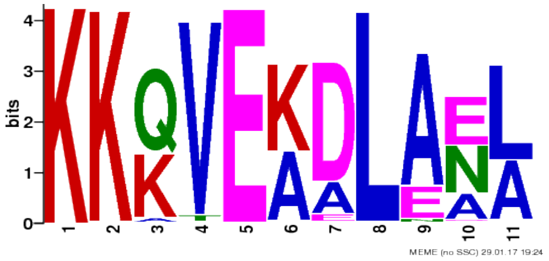

Motif	Logo
m07	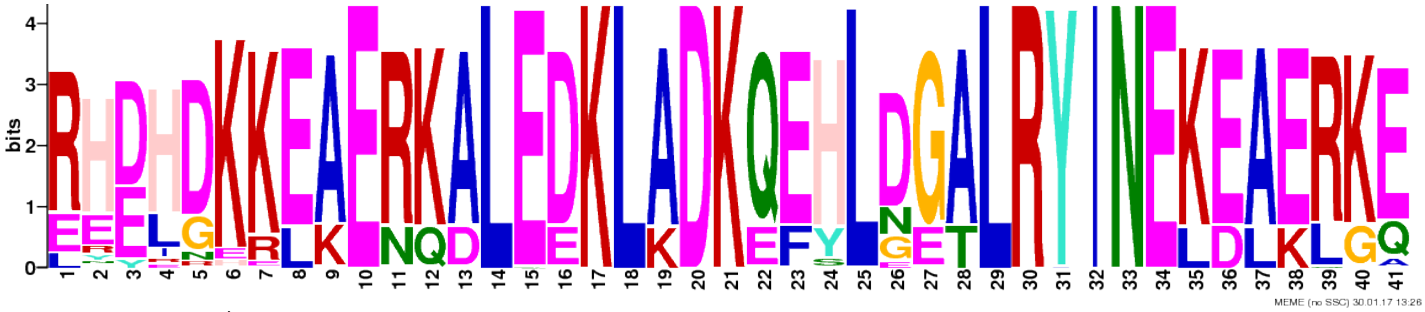
m08	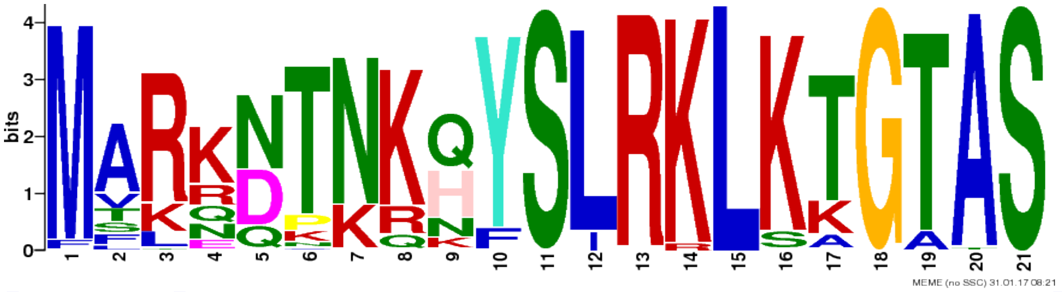
m09	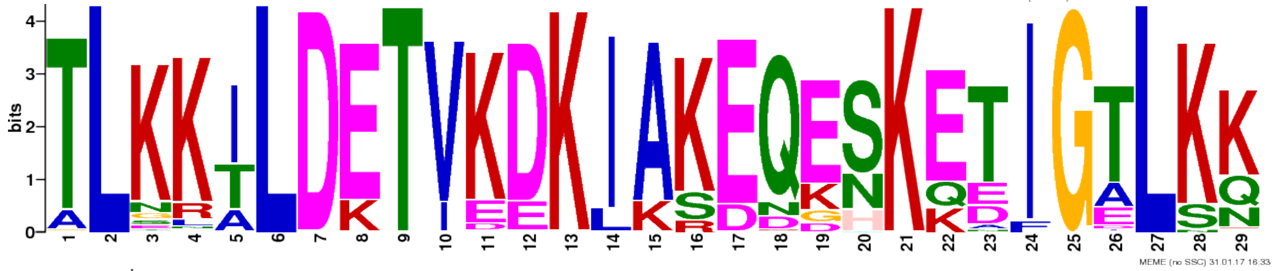
m10	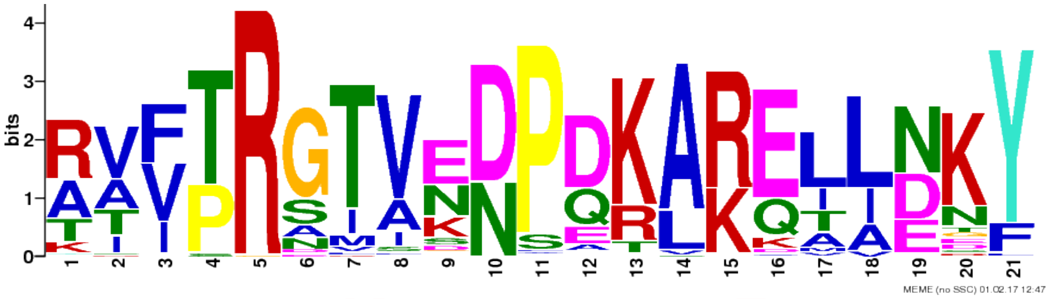
m11	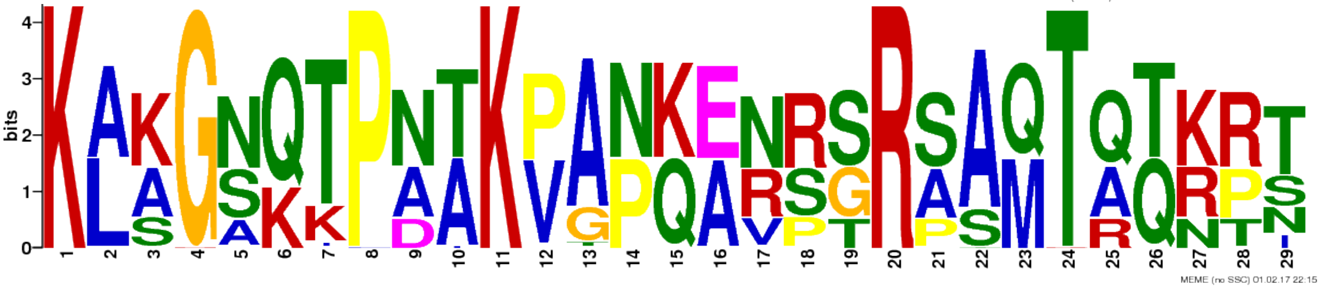
m12	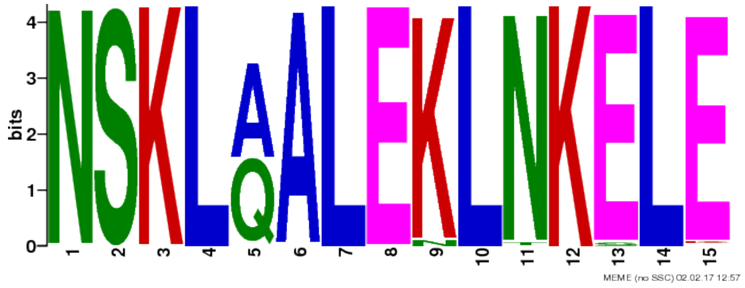

Motif	Logo
m13	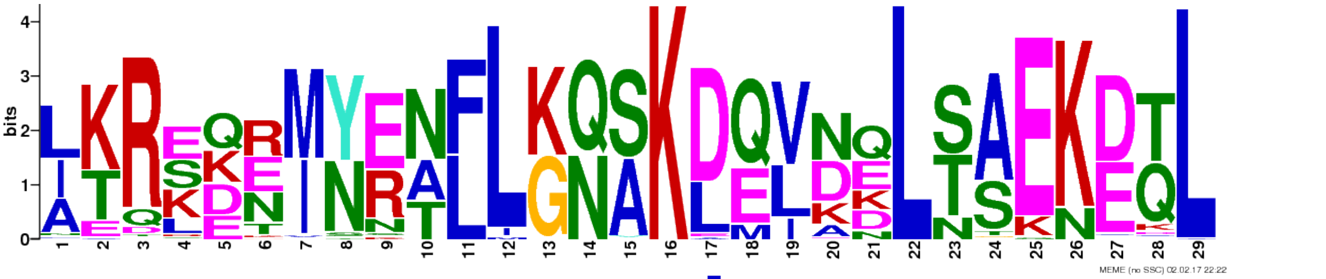
m14	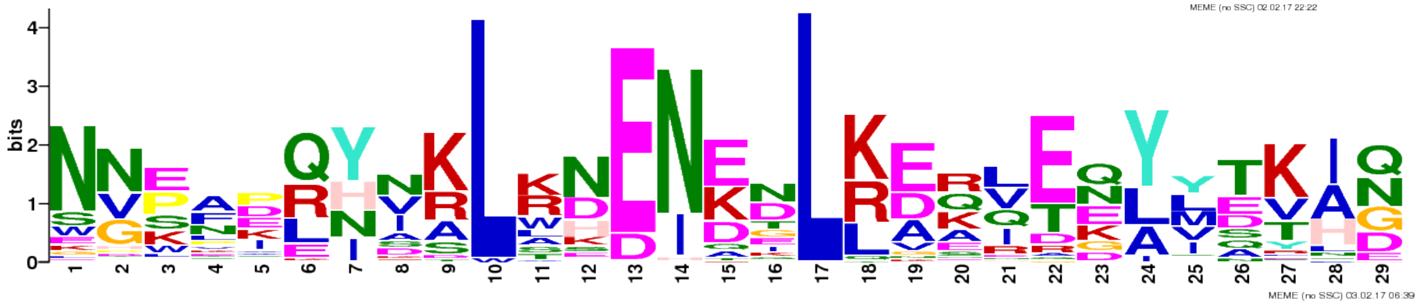
m15	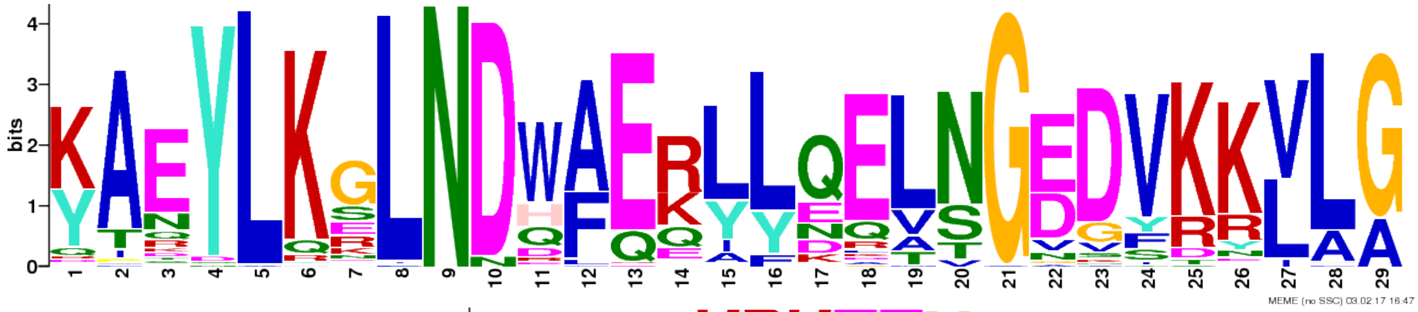
m16	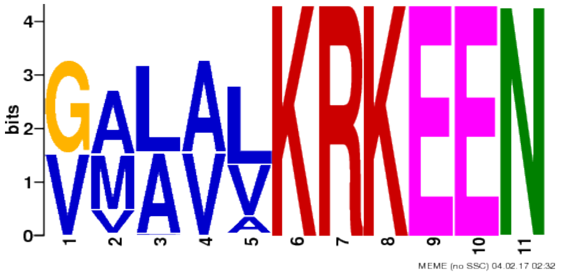
m17	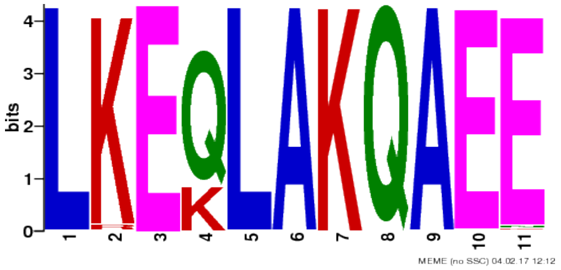
m18	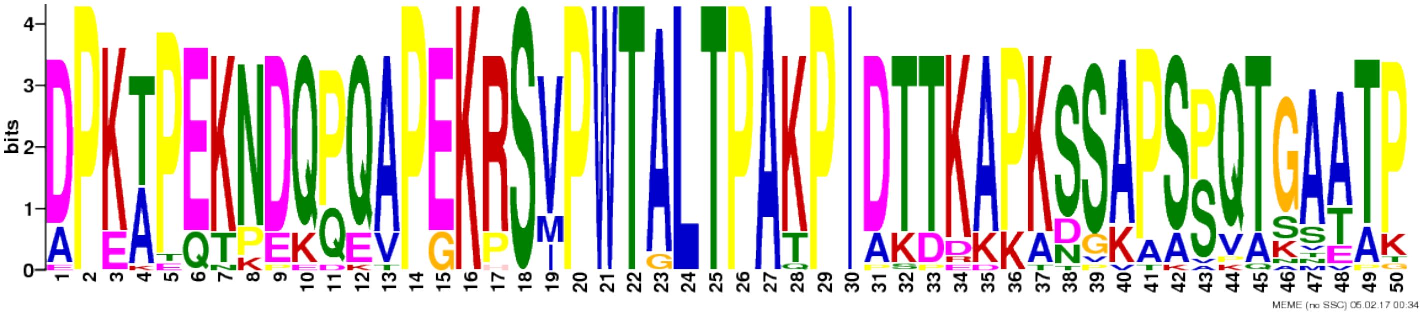

Motif	Logo
m19	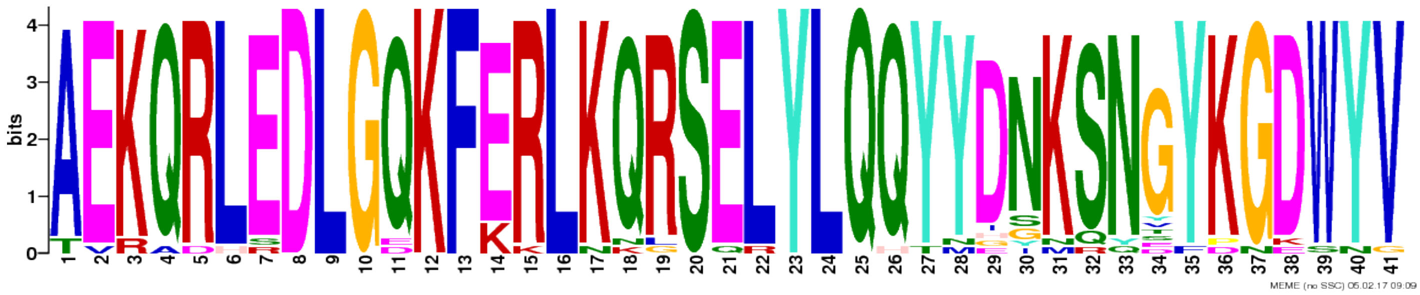
m20	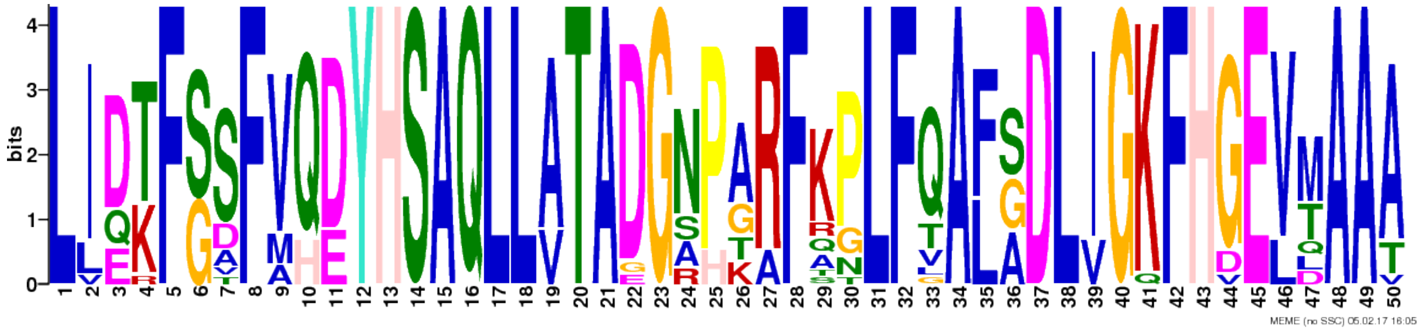
